# Resveratrol enhances prostate cancer cell response to ionizing radiation. Modulation of the AMPK, Akt and mTOR pathways

**DOI:** 10.1186/1748-717X-6-144

**Published:** 2011-10-26

**Authors:** Ayesha Rashid, Caiqiong Liu, Toran Sanli, Evangelia Tsiani, Gurmit Singh, Robert G Bristow, Ian Dayes, Himu Lukka, James Wright, Theodoros Tsakiridis

**Affiliations:** 1 Translational Radiation Biology Laboratory, Juravinski Cancer Centre, 699 Concession Street, Hamilton, Ontario, L84 5C2, Canada; 2 Department of Oncology, McMaster University, 1280 Main Street West, Hamilton, Ontario, L8S 4L8, Canada; 3 Department of Pathology and Molecular Medicine, McMaster University, 1280 Main Street West, Hamilton, Ontario, L8S 4L8, Canada; 4 Department of Community Health Science, Brock University, 500 Glenridge Avenue, St. Catharines, Ontario, L2S 3A1, Canada; 5 Department of Radiation Oncology, Princess Margaret Hospital, University of Toronto, 610 University Avenue, Toronto, Ontario, M5G 2M9, Canada

**Keywords:** radio-sensitizers, clonogenic survival, cell cycle, ATM, p53, p21^cip1^

## Abstract

**Background:**

Prostate cancer (PrCa) displays resistance to radiotherapy (RT) and requires radiotherapy dose escalation which is associated with greater toxicity. This highlights a need to develop radiation sensitizers to improve the efficacy of RT in PrCa. Ionizing radiation (IR) stimulates pathways of IR-resistance and survival mediated by the protein kinase Akt but it also activates the metabolic energy sensor and tumor suppressor AMP-Activated Protein Kinase (AMPK). Here, we examined the effects of the polyphenol resveratrol (RSV) on the IR-induced inhibition of cell survival, modulation of cell cycle and molecular responses in PrCa cells.

**Methods:**

Androgen-insensitive (PC3), sensitive (22RV1) PrCa and PNT1A normal prostate epithelial cells were treated with RSV alone (2.5-10 μM) or in combination with IR (2-8 Gy). Clonogenic assays, cell cycle analysis, microscopy and immunoblotting were performed to assess survival, cell cycle progression and molecular responses.

**Results:**

RSV (2.5-5 μM) inhibited clonogenic survival of PC3 and 22RV1 cells but not of normal prostate PNT1A cells. RSV specifically sensitized PrCa cells to IR, induced cell cycle arrest at G1-S phase and enhanced IR-induced nuclear aberrations and apoptosis. RSV enhanced IR-induced expression of DNA damage (γH2Ax) and apoptosis (cleaved-caspase 3) markers as well as of the cell cycle regulators p53, p21^cip1 ^and p27^kip1^. RSV enhanced IR-activation of ATM and AMPK but inhibited basal and IR-induced phosphorylation of Akt.

**Conclusions:**

Our results suggest that RSV arrests cell cycle, promotes apoptosis and sensitizes PrCa cells to IR likely through a desirable dual action to activate the ATM-AMPK-p53-p21^cip1^/p27^kip1 ^and inhibit the Akt signalling pathways.

## Introduction

Radiotherapy is an effective therapy for localized prostate cancer (PrCa) but this disease is highly resistant to ionizing radiation (IR). Conventional radiotherapy doses up to 70 Gy show biochemical failure rates of 30% or more in localized disease [1], leading to a need for RT dose escalation, which is associated with rectal and bladder toxicity. Therefore, there is a need for rational development of effective radiosensitizers for PrCa.

The phosphatidylinositol 3-kinase (PI3k)-Protein kinase B/Akt (henceforth: Akt) pathway is known to promote proliferation, cell cycle progression and resistance to cytotoxic therapies in PrCa [2]. PI3k is an effector of the epidermal growth factor receptor (EGFR) [3], that leads to recruitment of Akt and its activators to plasma membrane. Akt is activated by phosphorylation on residues T308 and serine S473, both of which are required for activation [4]. T308 phosphorylation is mediated by the phosphoinoisitide-dependent kinase 1 (PDK1) [5] but the kinase mediating S473 phosphorylation (PDK2) is not clearly defined. Candidate kinases include the DNA damage sensor Ataxia Telangiectasia Mutated (ATM) [6]. Activated Akt mediates transcription of genes involved in survival and inhibition of those involved in apoptosis (see [2,7] for review). It promotes cell cycle progression through inhibition of the cell cycle regulators p53 [8] and the cyclin-dependent kinase inhibitors (CDKI) p21^cip1 ^and p27^kip1 ^[9,10]. Furthermore, it regulates metabolic and nuclear processes through activation of the mammalian target-of-rapamycin (mTOR). Importantly, IR elicits cytoprotective responses mediated in part through activation of the PI3k-Akt pathway [11]. Akt is a mediator of radioresistance and PI3k-Akt pathway inhibitors are shown to enhance radiosensitivity of cancer cells [7,12].

AMPK is a heterotrimeric enzyme that consists of an α-catalytic and β-and γ-regulatory subunits [13]. It is a key regulator of carbohydrate and lipid metabolism and of proliferation in normal and cancer cells. AMPK detects an elevated AMP/ATP ratio in conditions of metabolic stress such as starvation and exercise [13] and promotes energy conservation by inhibiting protein synthesis, through mTOR inhibition while it also functions as a metabolic checkpoint to induce cell cycle arrest via p53 [14]. Recently, we showed that IR activates AMPK in human lung, breast and PrCa cells and suggested that AMPK participates in a signaling pathway involving ATM-AMPK-p53-p21^cip1 ^leading to regulation of the cell cycle and survival [15].

RSV (3,4',5-trihydroxystilbene) is a polyphenolic phytoalexin with widely reported anti-aging and anti-cancer properties [16,17]. It inhibits cancer cell proliferation and is suggested to enhance radiation responses [18,19]. RSV has also been reported to increase metabolic rate and reduce fat mass in wild-type mice but not in AMPK α subunit knockout mice [20]. Further, it was shown to suppress tumor growth and metastasis in the mouse Lewis lung carcinoma model [21]. RSV is known to regulate both Akt and AMPK [22,23] but the effects of this compound on the two signaling pathways have not been studied in radiated cells.

Here, we investigated the polyphenol RSV due to the reported ability of this natural compound to modulate both the radioresistance-mediating Akt and the tumour suppressor AMPK pathways [24,25].

## Materials and methods

### Cell Lines and Cell Culture

Human PrCa (PC3, 22RV1) and normal prostate epithelial (PNT1A) cell lines were obtained from American Tissue Culture Collection (Manassas, VA, U.S.A.). Cells were maintained at 37°C in RPMI media supplemented with 10% (vol/vol) Fetal Bovine Serum (FBS) and 1% (vol/vol) antibiotic-antimycotic (Invitrogen, Burlington, ON, Canada).

### Reagents and Antibodies

Rabbit polyclonal antibodies against total Akt (T-Akt), phosphorylated-(P)-(S473)-Akt, P-(T308)-Akt, P-mTOR, total AMPK (T-AMPK), P-(Thr172)-AMPK, T-ATM, P-(S1981)-ATM, P-(S139)-γH2Ax, mouse monoclonal antibodies against p53, p21^cip1^, p27^kip1^, actin, an anti-α-tubulin antibody conjugated to Alexa Fluor 488 as well as horseradish peroxidase (HRP)-conjugated IgG secondary anti-rabbit and anti-mouse antibodies were from New England Biolabs (Mississauga, ON, Canada). Hoechst 33258 was from Sigma (Toronto, ON, Canada). RSV and the KuDos Pharma ATM inhibitor KU55933 were from Calbiochem (Mississauga, ON, Canada). Anti-α1 and -α2 AMPK siRNA transfection kit was obtained from Qiagen (Mississauga, Ontario, Canada).

### Treatments

Cells were treated with 2-8 Gy IR using a ^60^Co clinical unit. For combined RSV or KU55933 and IR treatments, cells were kept at 37°C with the indicated agent for 1 h prior to IR treatment. Cells were incubated for 1 h following IR exposure, unless otherwise indicated. For cell cycle and clonogenic assays cells were exposed to the treatment agents throughout the experiments (as indicated in figure legends).

### siRNA AMPK α Subunit Knockdown

Cells were incubated with a mixture of human siRNA sequences against the α1 and α2 AMPK subunits using HiPerFect vehicle for 72 hours as per the manufacturer's protocol.

### Clonogenic Assays

Clonogenic assays were performed as described earlier [15]. Cells (500-1000) were seeded in triplicates and allowed to adhere overnight, then were incubated with RSV followed by IR treatment (0-2 Gy) followed by incubation for 7-10 days. Cells were then fixed and stained with 0.05% methylene blue and colonies (> 50 cells) were counted. Results are expressed as the surviving fraction compared to untreated control.

### Immunoblotting (IB)

IB was performed as described earlier [26]. Approximately 5 × 10^5 ^cells were seeded in 6-well plates. After treatment, cells were washed, lysed and twenty micrograms of protein were subjected to IB.

### Immunofluorescence Microscopy

Cells (2 × 10^5^) were seeded onto glass coverslips and were incubated without or with RSV (5 μM) 1 h prior to IR treatment (8 Gy). Ninety six hours after IR exposure, cells were washed lightly with PBS and fixed with 3% paraformaldehyde/PBS/0.2% Triton X-100 for 20 minutes. Cells were stained with Hoechst 33258 (20 μM) nuclear stain or anti-α-tubulin antibody conjugated to Alexa Fluor 488 (1:100 dilution) (followed by Hoechst 33258 nuclear staining) and examined by fluorescence microscopy. Four hundred cells were evaluated for nuclear aberrations (fragmentation, micro-nuclei and multi-nucleation) in four representative areas of each slide in 3 independent experiments.

### Cell Cycle Analysis

(1 × 10^6 ^cells/mL) were seeded in 10-cm dishes. After treatment, cells were trypsinized, washed, fixed with 70% ethanol and stored overnight at -20°C followed by washing and staining with a solution containing 100 μL Triton X-100 and 50 μg/mL propidium iodide. Cells were subjected to flow cytometric cell cycle analysis using a Beckman Coulter Epics XL flow cytometer.

### Statistical Analysis

Data are expressed as the mean ± standard error (SE). Statistical analysis was performed using unpaired t-test with SPSS v16.0 software (SPSS, Chicago, IL). Statistical significance was considered at p < 0.05.

## Results

### RSV inhibits PrCa cell survival

**Figure 1:**
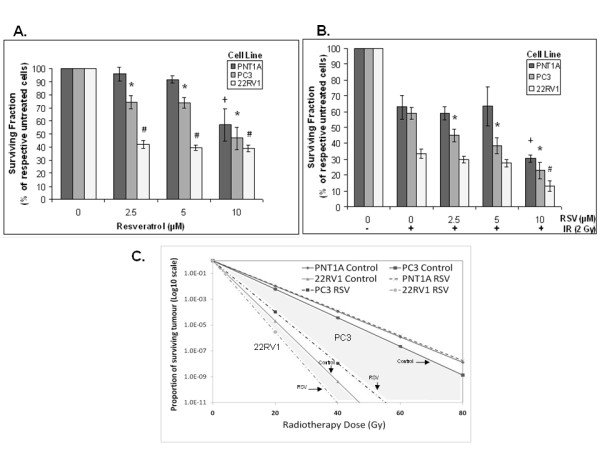
**Effects of resveratrol (RSV) and ionizing radiation (IR) on survival of prostate cancer (PrCa) cells**. **A**. Cells were incubated with increasing doses of RSV (0-10 μM) and allowed to form colonies. Colonies (> 50 cells) were counted. Results are expressed as the surviving fraction compared to untreated control (see *Methods)*. '*' p < 0.05 compared to untreated PC3 cells, '#' p < 0.05 compared to untreated 22RV1 cells, '+' p < 0.05 compared to untreated PNT1A cells. **B**. Cells were pre-treated with RSV (0-10 μM) followed by treatment with a single fraction of IR (2 Gy) and allowed to grow and form colonies. Treatment values were normalized to untreated controls and surviving fractions are presented as percent of control. Shown is the mean of 4-5 independent experiments ± standard error (SE). '*' p < 0.05 compared to 2 Gy treated PC3 cells, '#' p < 0.05 compared to 2 Gy treated 22RV1 cells, '+' p < 0.05 compared to 2 Gy treated PNT1A cells. **C**. Projected proportion of tumour survival after treatment with 0 - 80 Gy was estimated using the Surviving Fraction at 2 Gy (SF2) values for control and RSV (5 μM) treated PNT1A, PC3 and 22RV1 cells. SF2^n ^was determined as the tumour proportion surviving after "n" 2 Gy fractions, assuming iso-effectiveness of each fraction. Values were plotted up to a scale of 1 × 10^-11 ^to reflect elimination of tumour of 50 - 100 gr expected to contain 5 - 10 × 10^10 ^cells.

As a single agent, RSV effectively inhibited survival of both PC3 and 22RV1 PrCa cells with significant inhibition achieved at even the lower doses of 2.5 and 5 μM (p < 0.05) (Figure 1A). The IC_50 _values were approximately 10 μM and 2.5 μM for PC3 and 22RV1 cells, respectively. The PC3 response to RSV was dose-dependent. RSV 2.5 μM significantly decreased survival in 22RV-1 cells to 40 ± 3.06% of control without any further decrease at higher doses. In contrast, PNT1A normal epithelial cells were less responsive to RSV showing only 5 and 10% inhibition of survival at 2.5 and 5 μM, respectively (Figure 1A). Only the higher dose of 10 μM caused significant decrease in normal epithelial PNT1A cell survival (to 60 ± 12.26% of control) (p < 0.05). PTN1A cells were used here as a non-malignant control.

### IR-induced inhibition of PrCa cell survival

PC3 cells showed greater resistance to IR alone compared to 22RV1 cells (surviving fractions after 2 Gy (SF2) of 60 ± 5.30% vs 40 ± 3.53%, respectively, Figure 1B). Their IR sensitivity was similar to that of PNT1A cells (SF2 of 62.9 ± 2.26%, Figure 1B). In clonogenic assays we opted to use the standard therapeutic IR dose of 2 Gy and not high doses of IR, for clinical relevance and because higher doses (i.e. 6-8 Gy) were highly toxic to PrCa cells in agreement with previous reports [27].

### RSV enhances the IR-induced inhibition of clonogenic survival in PrCa cells

RSV augmented further the IR-induced inhibition of survival in both PC3 and 22RV1 PrCa cells. RSV (2.5 and 5 μM) decreased survival of IR-treated PC3 cells by (13.8 ± 0.09% and 20.4 ± 0.9%, Figure 1B). RSV 5 μM caused only a minor reduction of SF2 in 22RV1 cells (non-significant) but at 10 μM the drug induced a significant 2.5-fold inhibition in SF2. RSV (5 μM) pre-treatment did not decrease further SF2 of PNT1A prostate epithelial cells but this was achieved when RSV was increased to 10 μM (Figure 1B).

To better demonstrate the potential clinical benefit of RSV in combination with radiotherapy, we used SF2 values to estimate tumour cell survival after 40 fractions of radiotherapy (2 Gy each), which consists a standard approach in clinical PrCa management [1]. We determined (SF2)^n ^as the tumour cell fraction surviving after "n" fractions of radiotherapy. Assuming iso-effectiveness of all fractions, we plotted the projected tumour survival over 40 consecutive fractions (0-80 Gy) (Figure 1C). As typical human prostates range between 35-100 gr (5 - 10 × 10^10 ^cells) we plotted projected tumour size reductions (tumour killing) on a scale of 1 - 1 × 10^-11^. Figure 1C estimates that RSV (5 μM) may be able to reduce the dose of radiotherapy needed to eliminate 22RV1 type (hormone-sensitive) PrCa tumours (from about 47 to 40 Gy) and may be able to eliminate PC3 type (hormone-insensitive) PrCa tumours (with about 54 Gy) that would otherwise be incurable with doses even higher than 80 Gy. Further, this approach also demonstrated the complete lack of radio-sensitization of normal epithelial cells (PNT1A) by 5 μM RSV. Based on these results, we pursued all subsequent studies with PrCa cells alone and with doses of 2.5 - 5 μM RSV.

### RSV inhibits IR-mediated cell cycle arrest and induces cell accumulation at G1-S and sub-G1 phases consistent with apoptosis

**Figure 2:**
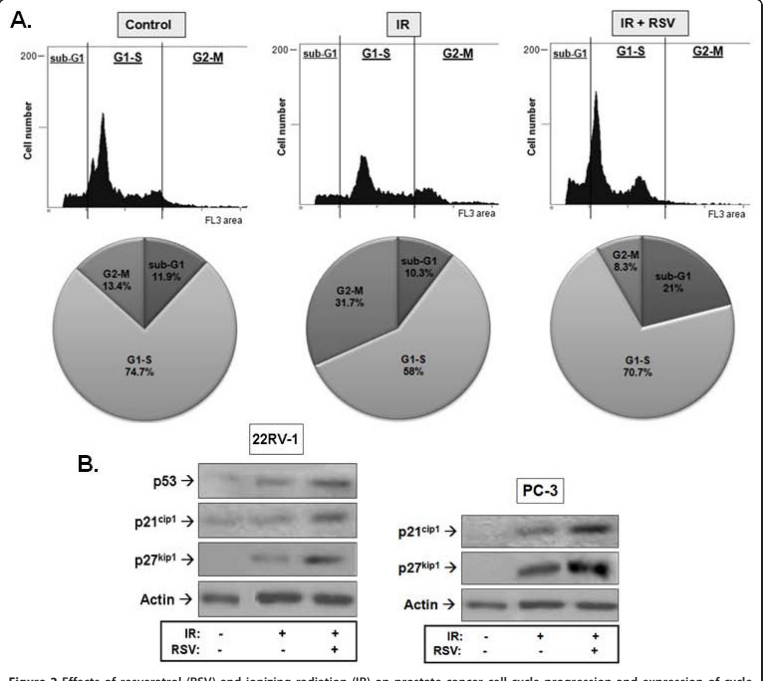
**Effects of resveratrol (RSV) and ionizing radiation (IR) on prostate cancer cell cycle progression and expression of cycle regulators**. **A**. 22RV1 cells were pre-treated or not with RSV (5 μM) for 1 hour followed by treatment with 8 Gy IR. Cells were allowed to cycle for 48 hours after which they were fixed, treated with a triton-X and stained with propidium iodide (PI) solution as described in *Methods*. Shown are representative results of 3 independent experiments. Pie charts illustrate the proportion of cells in each phase of cell cycle. **B**. 22RV1 and PC3 cells were pre-treated with RSV (5 μM) for 1 hour followed by 8 Gy IR treatment. Cells were lysed 1 hour after IR exposure and subjected to immunoblot analysis using antibodies against the indicated cell cycle markers. Shown are representative immunoblots of 3 independent experiments.

We utilized the 22RV1 cell line in our cell cycle analysis experiments because it is a cloned cell line [28] consisting of a more isogenic population of cells than PC3 cells, and is hence appropriate for cell cycle analysis. As expected, IR induced an arrest of cells at the G2-M interphase (Control: 13.4% vs. IR: 31.7%, Figure 2) but pre-treatment with RSV (5 μM) prevented this event (RSV+IR: 8.3% vs. 31.7% for IR, Figure 2). RSV caused accumulation of radiated cells into the G1-S phases of the cycle (IR: 58% vs. RSV + IR: 70.7%) and increased the population of cells in the sub-G1 region by 2-fold compared to those treated with IR alone (IR: 10.3% vs. RSV + IR: 21.0%), indicating induction of apoptosis.

### RSV enhances the IR induction of cell cycle inhibitors

To examine whether the effects of RSV on IR regulation of the cell cycle were associated with enhancement of molecular pathways of cell cycle regulation, we examined the expression of p53 and the cyclin-dependent kinase inhibitors (CDKI) p21^cip1 ^and p27^kip1^. Figure 2B shows that IR induced expression of both p53 and the CDKIs in 22RV1 cells and that RSV enhanced this further. Similar results were obtained for the two CDKIs in p53-null PC3 cells.

### RSV induces expression of apoptotic markers and nuclear aberrations

**Figure 3:**
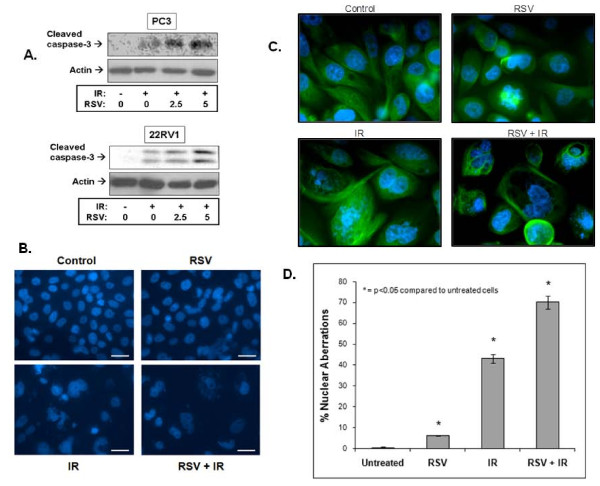
**Resveratrol (RSV) enhances ionizing radiation (IR)-induced cell death**. Induction of cleaved caspase-3 and nuclear aberrations. **A**. PC3 cells were pre-treated or not with RSV (5 μM) for 1 hour after which they were treated with IR (8 Gy). Cells were lysed 1 hour after IR exposure and subjected to immunoblot analysis using the indicated antibodies. Shown are representative immunoblots of 3-4 independent experiments. **B**. and **C**. Cells were treated or not with RSV (5 μM) prior to IR treatment (8 Gy) followed by staining with nuclear stain Hoeschst 33258 (low magnification, in **B**) or Hoeschst 33258 and immunostaining of tubulin (high magnification, in **C**) used to illustrate better cellular morphology. Morphology was assessed using fluorescence microscopy. Representative images from 4 independent experiments are shown. **D**. Average ± SE values (% Nuclear Aberrations: as indicated by the proportion of cells with features of nuclear fragmentation, micro-nuclei and multi-nucleation) were extracted through quantitation of 4 independent experiments (in **B**), as described in *Methods*.

Using immunoblotting with a cleaved caspase-3-specific antibody, we examined the effects of RSV and IR on the levels of this established apoptosis marker. IR increased caspase-3 cleavage within 1 hour of IR exposure in both PC3 and 22RV1 cells (Figure 3A). Pre-treatment with RSV (2.5 and 5 μM) enhanced the IR-induced cleaved caspase-3 levels in a dose-dependent fashion.

To evaluate morphological markers of apoptosis, cells were stained with Hoechst 33258 and examined with microscopy. Cells with nuclear aberrations such as fragmentation, micro-nuclei and multi-nucleation (polysomy), which are markers predictive of apoptosis and/or mitotic cell death (mitotic catastrophe), were counted in each treatment condition. Figure 3B shows representative images of treated PC3 cells that were either stained with Hoechst 33258 alone (low magnification, Figure 3B) or additional immunostaining of tubulin (high magnification), which was utilized to better demonstrate the effects of the treatments on nuclear and cellular morphology (Figure 3C). The results of four independent experiments (as in Figure 3B) were quantitated (as described in *Methods*) and are shown in Figure 3D). IR alone induced significant nuclear aberrations (43 ± 2.18%, Figure 3B), while RSV alone (5 μM) increased nuclear abnormalities by 6% (Figure 3B). However, RSV significantly enhanced the IR-induced nuclear aberrations to 70 ± 3.11% (an increase of 27%, Figure 3D) of total cell counts and reduced overall cellular viability (Figure 3B).

### Effects of RSV on molecular pathways of radio-resistance and tumour suppression

#### RSV inhibits basal and IR-induced levels of phosphorylated Akt

**Figure 4:**
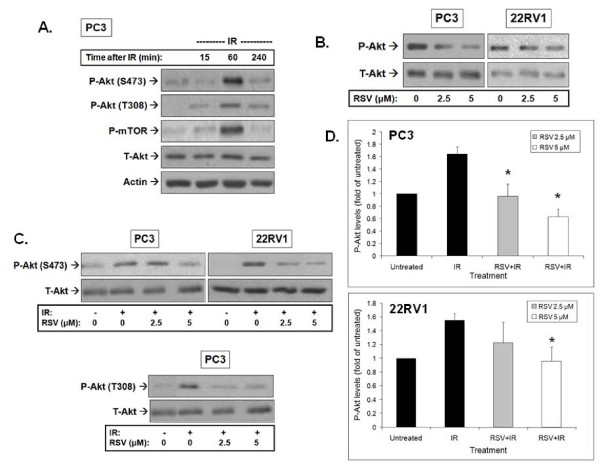
**Resveratrol (RSV) inhibits basal and ionizing radiation (IR)-induced Akt phosphorylation. A**. Effects of IR alone: PC3 cells were treated with 0 or 8 Gy IR and lysed according to the indicated time points following IR exposure. Then cells were subjected to immunoblot analysis. **B**. RSV inhibits basal Akt phosphorylation: PC3 and 22RV-1 cells were treated with RSV (0, 2.5 or 5 μM) for 1 hour and subjected to lysis and immunoblot analysis using the indicated antibodies. **C**. RSV inhibits IR-induced Akt phosphorylation. PC3 and 22RV-1 cells were pre-treated with the indicated concentrations of RSV 1 hour prior to IR treatment. Cells were lysed 1 hour after IR exposure and subjected to immunoblot analysis. Shown are representative immunoblots of 4 independent experiments. **D**. Densitometric quantitation of Akt (S473) phosphorylation and total Akt was performed. P-Akt was normalized to T-Akt levels and presented as fold change compared to untreated control cells (mean ± SE) from 3-4 independent immunoblotting experiments as in **C**). * p < 0.05 compared to cells treated with IR alone.

We examined the effects of RSV on the well-described radio-resistance pathway of Akt-mTOR. We observed that IR alone induced a significant time-dependent increase in the levels of phosphorylated Akt (P-Akt) (both S473 and T308 sites) in PC3 cells, with no effects on total levels of the protein (Figure 4A). Highest levels of P-Akt were reached 30 minutes to 1 hour after IR exposure. Akt phosphorylation was associated with increased activity of this enzyme indicated by the IR-induced phosphorylation of mTOR, a key effector of Akt (Figure 4A). Similar results were obtained in 22RV1 PrCa cells (results not shown).

Treatment of PC3 and 22RV1 cells with RSV inhibited basal levels of P-Akt (Figure 4B). Of note, the images shown were obtained after significantly longer film exposure to allow demonstration of the effects of RSV on basal Akt phosphorylation which are significantly lower than in radiated cells (as shown in Figure 4A and 4C). Pre-treatment with RSV led to significant inhibition of IR-induced P-Akt with no effects on total levels of the protein (Figure 4C). Although IR exposure led to an increase in P-Akt, RSV pre-treatment (2.5 and 5 μM) significantly reduced and/or prevented IR-induced phosphorylation of Akt. The effects of RSV on IR-induced Akt phosphorylation on S473 were quantitated in four independent experiments (Figure 4D). Similar results were obtained for Akt T308 phosphorylation. A representative immunoblot from PC3 cells is shown (Figure 4C).

#### RSV and IR effects on AMPK

**Figure 5:**
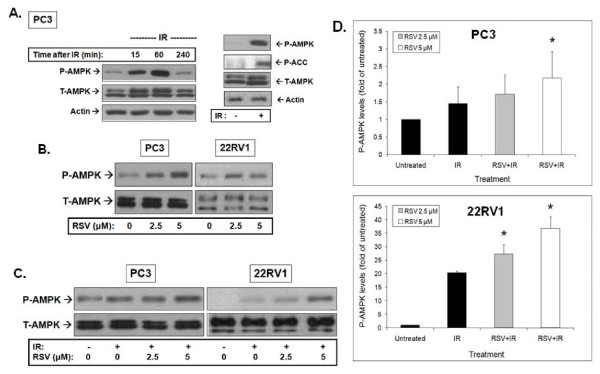
**Resveratrol (RSV) enhances basal and ionizing radiation (IR)-induced AMPK phosphorylation**. **A**. Effects of IR alone: PC3 cells were treated with 0 or 8 Gy IR and lysed at the indicated time points after IR exposure. The adjacent panel shows PC3 cells treated with 8 Gy IR, which were lysed 1 hour after IR treatment. Samples were subjected to immunoblot analysis using the indicated antibodies. **B**. RSV enhances basal AMPK phosphorylation: PC3 and 22RV-1 cells were treated with the indicated concentrations of RSV for 1 hour followed by lysis and immunoblotting analysis. **C**. RSV enhances IR-induced AMPK phosphorylation. PC3 and 22RV-1 cells were pre-treated with the indicated concentrations of RSV 1 hour prior to 8 Gy IR treatment. Cells were lysed 1 hour after IR exposure and subjected to immunoblotting using indicated antibodies. Shown are representative immunoblots of at least 3-4 independent experiments. **D**. Densitometry-based quantitation. Density values of P-AMPK were normalized to T-AMPK levels and presented as fold change compared to untreated control cells (mean ± SE) from 2-3 independent experiments in **C**. is shown. * p < 0.05 compared to cells treated with IR alone.

We reported earlier that IR activates AMPK in lung, breast and prostate cancer cells [15]. Similarly, we observed here that IR induced a robust phosphorylation of AMPK on Thr172 of the catalytic α subunit that was detectable within 15 min. Highest levels of phosphorylated AMPK (P-AMPK) were detected 1 hour after IR exposure and returned to almost basal levels 4 hours after radiation (Figure 5A). This was associated with activation of AMPK indicated by the detected phosphorylation of the AMPK substrate Acetyl CoA Carboxylase (ACC). RSV induced a robust activation of AMPK in both PC3 and 22RV1 cells at both 2.5 and 5 μM (Figure 5B). Furthermore, RSV pre-treatment enhanced significantly the IR-induced AMPK phosphorylation in both 22RV1 and PC3 cells (Figure 5C). Total AMPK levels remained unchanged after both IR and RSV treatments. The results of 3-4 independent experiments were quantitated and are shown in Figure 5D.

### The pathway of radiation activation of Akt and AMPK

#### Akt regulation

**Figure 6:**
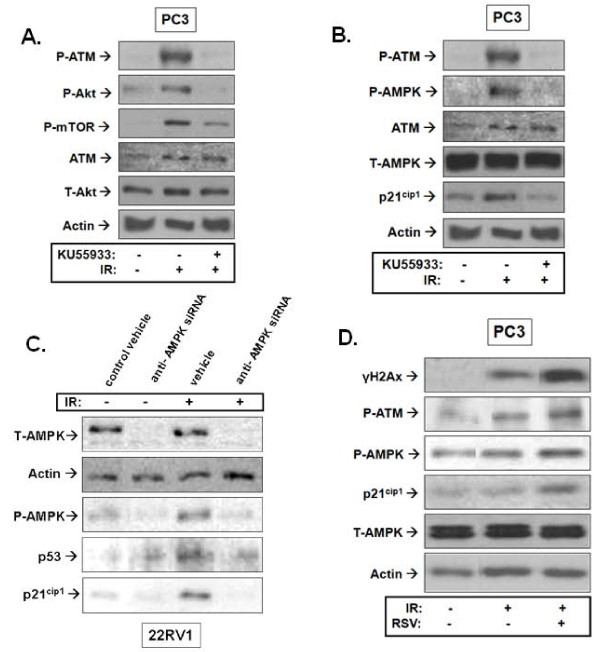
**Mechanism of Akt and AMPK regulation by resveratrol (RSV) and ionizing radiation (IR)**. **A**. and **B**. Inhibition of ATM blocks IR-induced activation of the Akt and AMPK signaling pathways: PC3 cells were treated or not with the ATM inhibitor KU55933 (10 μM) for 1 hour followed by 0 or 8 Gy IR treatment. Cells were lysed 1 hour after IR exposure and subjected to immunoblot analysis using the indicated antibodies. **C**. AMPK mediates the IR induction of p53 and p21^cip1^: 22RV-1 cells were incubated with siRNA complexes against the α1 and α2 subunits of AMPK for 72 hours as described in *Methods*. Cells were then treated with 0 or 8 Gy IR and lysed 1 hour after IR exposure and subjected to immunoblot analysis. **D**. RSV enhances signalling along the ATM-AMPK-p21^cip1 ^pathway: Cells were pre-treated with RSV (5 μM) for 1 hour followed by IR of 8 Gy. Cells were lysed 1 hour after IR exposure and subjected to immunoblot analysis using the indicated antibodies. Shown are representative immunoblots of at least 3 independent experiments.

Recent reports suggest that Akt activation by IR may be regulated by ATM [29]. We examined this notion in PrCa cells using the ATM-specific inhibitor KU55933 [30]. Pre-incubation with KU55933 prevented IR-induced ATM phosphorylation but also inhibited IR phosphorylation of Akt at S473 and activation of its kinase activity as indicated by reduced phosphorylation of mTOR (Figure 6A).

#### AMPK Regulation

We attempted to verify, in PrCa cells, our earlier observations in lung cancer cells [15] of AMPK participation in an ATM-AMPK-p53/p21^cip1 ^pathway activated by IR. We observed a robust phosphorylation of ATM and AMPK as well as induction of p21^cip1 ^in PC3 PrCa cells in response to IR. IR induced ATM and AMPK phosphorylation and p21^cip1 ^induction were all inhibited by treatment with KU55933 (Figure 6B). To verify that AMPK truly acts as a mediator of p53/p21^cip1 ^induction in response to IR in PrCa cells, we used wild-type p53 expressing 22RV1 cells to perform AMPK knockdown experiments. siRNA knockdown of both α1 and α2 subunits of AMPK blocked p53 and p21^cip1 ^induction by IR (Figure 6C). Interestingly, RSV pre-treatment enhanced IR-induced phosphorylation of ATM and of its substrate histone H2Ax (γH2Ax), as well as phosphorylation of AMPK and induction of p21^cip1^.

## Discussion

### IR and RSV effects on PrCa cell clonogenic survival

RSV inhibits survival and proliferation of cancer cells as a single agent and induces radiosensitization in human cervical cancer cells [19]. Similarly, we observed that at low doses (2.5-10 μM) RSV inhibited clonogenic survival of PrCa cells. Several studies have reported IC_50 _values for cell growth inhibition by RSV in the range of 5 to 10 μM [31,32]. Free RSV has a low bioavailability *in-vivo *as it is rapidly metabolized to glucoronide and sulfate conjugates [32]. A human study reported plasma concentrations of free RSV of 21 nM after oral dose of 25 mg RSV [32]. However, all combined RSV metabolites were reported to reach about 2 μM [16]. For this, we pursued our studies with low RSV concentrations (2.5 - 10 μM).

As a single agent, RSV inhibited more potently survival of 22RV1 compared to PC3 cells but the response of 22RV1 cells did not display dose-dependence. Overall, PC3 cells displayed greater resistance to both IR and RSV alone, consistent with other studies [33]. PC3 cells are deficient in key tumour suppressors including PTEN and p53 [34]. Lack of PTEN allows aberrant Akt activation [35], which in combination with the lack of p53, may confer such cells a survival advantage and IR- and RSV-resistance. PNT1A cells were less responsive to RSV, indicating a potential for this drug to achieve a positive therapeutic ratio *in-vivo*. RSV inhibited significantly PNT1A cells at 10 μM and for that we focused our work on 2.5 and 5 μM RSV.

We demonstrated that RSV can sensitize PrCa cells to IR (Figure 1). Concentrations of RSV (2.5 and 5 μM), similar to those that can be achieved in human serum, enhanced the cytotoxicity of a conventional RT fraction (2 Gy) in PrCa cells without additional toxicity to normal epithelial cells. The potential clinical utility of our finding is illustrated in Figure 1C, which suggests that low RSV doses have the potential to reduce the dose of radiotherapy required to treat human hormone- and radiation-sensitive PrCa and may be able to make curable hormone- and radiation-insensitive tumours that may otherwise be incurable with even modern dose escalated radiotherapy. This notion needs to be verified in *in-vivo *models of human PrCa.

### Regulation of cell cycle and apoptosis

RSV is reported to arrest PrCa and other cells at G0-G1 and/or S-phases of the cycle leading to senescence [36,37] and cause p21^cip1^-mediated G1-phase arrest and apoptosis in A431 cells [38]. Consistently, we observed a significant arrest of radiated PrCa cells at G1-S (Figure 4). RSV's effects on survival were additive to those of IR but the two agents mediate different regulation of cell cycle. Whereas IR induces G1-S and more so G2-M cycle arrest, RSV prevented the latter (Figure 2), likely due to induction of an earlier checkpoint (G1-S). The potentiation of IR-induced expression of p53 and CDK inhibitors p21^cip1 ^and p27^kip1 ^by RSV (Figure 2B), known to regulate the G1 and S phase checkpoints, may provide a molecular pathway of action for RSV's induction of the G1-S phase arrest seen in our studies.

RSV also caused re-distribution of cells into the sub-G1 or apoptotic range that was associated with cleavage of caspase-3 and induction of extensive nuclear aberrations (Figure 3). Apoptosis appears to be the primary mode of cell death induced by RSV. Studies demonstrated such cell death effects in PrCa cells through activation of caspase-, p53-, or Fas ligand-dependent pathways [39,40]. In our studies, IR-mediated nuclear damage showed early signs of mitotic catastrophe and RSV potentiated such nuclear aberrations (Figure 3C-D). Mitotic cell death predominates in cells with defects in cycle checkpoints that prevent cycle arrest and DNA repair when cells are exposed to genotoxic stress such as IR [41]. The features of mitotic catastrophe seen in our studies in PC3 cells (p53-*null*) after RSV and IR treatments are consistent with those observed earlier in other p53-*null *cancer models [41].

### Modulation of the Akt and AMPK pathways

IR and RSV exert opposite effects on Akt. IR mediated Akt phosphorylation on T308 and S473 and enzymatic activation indicated by mTOR phosphorylation (Figure 4A). This suggests activation of both PDK1 and PDK2 activity by IR. However, we observed that RSV is an effective inhibitor of this radioresistance-associated pathway (Figure 4). Inhibition of IR-induced T308 and S473 phosphorylation by RSV suggests an ability to regulate both PDK1 and PDK2. The enhancement of IR-induced cell death by RSV may indeed be mediated through modulation of Akt and its downstream targets as suggested earlier for uterine cancer [25] and PrCa cells [42].

Using the ATM-specific inhibitor KU55933, [30] we observed that ATM mediates IR-induced Akt S473 phosphorylation and activation, suggesting that ATM may indeed be the IR responsive PDK2, as suggested earlier [6]. However, despite inhibition of Akt, RSV enhanced ATM phosphorylation and activation indicated by histone H2Ax phosphorylation (γH2Ax) (Figure 6D). This points to a complex regulatory action of RSV to enhance ATM activation but inhibit its PDK2 action on Akt downstream. This concept needs to be explored further in future studies.

We aimed to evaluate, in PrCa cells, the DNA damage responsive ATM-AMPK-p21^cip1 ^pathway we proposed earlier in lung cancer cells [15] and found that IR indeed mediates AMPK activation downstream of ATM (Figure 6B). We observed that AMPK mediates p53 and p21^cip1 ^induction in response to IR in PrCa cells (Figure 6C). RSV and IR stimulated phosphorylation/activation of AMPK, in both PC3 and 22RV1 PrCa cells, which was associated with induction of p21^cip1 ^and p27^kip1 ^(Figure 6D). Although, we did not examine whether the latter is regulated by AMPK activity, this notion is well described by other studies [43]. The enhancement of IR-stimulated ATM activity by RSV provides a framework for upregulation of the ATM-AMPK-p21^cip1 ^pathway in RSV-treated cancer cells and a rationale for the observed inhibition of cell cycle and survival. This notion is supported by observations that the AMPK inhibitor compound C inhibits IR-induced cytotoxicity in PrCa (results not shown).

## Conclusions

**Figure 7:**
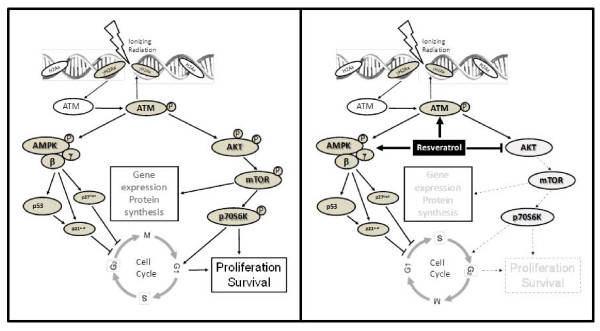
**Model of the mechanism of action of resveratrol (RSV) and ionizing radiation (IR) to regulate cell cycle and survival in PrCa cells**. **A**. IR induces DNA strand breaks which are detected by ATM, leading to AMPK activation and induction of the cell cycle inhibitors p53, p21^cip1 ^and p27^kip1^. IR also activates Akt to increase protein synthesis through mTOR and stimulate cell cycle progression, proliferation and survival. **B**. The work presented here suggests that RSV i) enhances the effects of IR on AMPK activation, leading to an early cell cycle arrest, and ii) inhibits Akt to reduce gene expression and proliferation and enhance radiation cytotoxicity.

Figure 7 illustrates a model of the effects of RSV on the IR responses of PrCa cells. Low (μM) concentrations of RSV, that can be achieved in human serum, inhibit PrCa cell survival and enhance IR-induced cytotoxicity. RSV enhances IR-induced DNA-damage response signals (ATM), apoptosis markers (cleaved caspase-3) and cell cycle inhibitors (p53, p21^cip1 ^and p27^kip1^). Further, it arrests cycling at the G1-S phase and enhances IR-induced nuclear aberrations. RSV exerts a desirable dual action to inhibit the Akt radioresistance pathway while enhancing the tumour suppressor AMPK, leading to potentiation of an ATM-AMPK-p53-p21^cip1 ^/p27^kip1 ^signaling axis. We conclude that RSV is a promising agent that deserves further investigation as an adjunct to IR in *in-vivo *models of PrCa to help elucidate its potential for clinical use in combination with RT.

## Competing interests

The authors declare that they have no competing interests.

## Authors' contributions

AR carried out the cellular/molecular experiments (clonogenic assays), cell cycle studies and helped draft the manuscript. CL aided in the cell cycle studies. TS helped carry out the immunofluorescence experiments. TT conceived the study, directed the study design, supervised all experimental work and prepared the manuscript. ET, GS, RB participated in the design of the study and advised TT on experimental approaches. All authors have read and approved the final manuscript.
